# Liver Involvement in Congenital Disorders of Glycosylation and Deglycosylation

**DOI:** 10.3389/fped.2021.696918

**Published:** 2021-07-05

**Authors:** Patryk Lipiński, Anna Bogdańska, Piotr Socha, Anna Tylki-Szymańska

**Affiliations:** ^1^Department of Pediatrics, Nutrition and Metabolic Diseases, Children's Memorial Health Institute, Warsaw, Poland; ^2^Department of Biochemistry, Radioimmunology and Experimental Medicine, Children's Memorial Health Institute, Warsaw, Poland; ^3^Department of Gastroenterology, Hepatology, Feeding Difficulties and Pediatrics, Children's Memorial Health Institute, Warsaw, Poland

**Keywords:** congenital disorder glycosylation, liver disease, hepatomegaly, coagulopathy, elevated serum transaminases, NGLY1-conegnital disorder of deglycosylation

## Abstract

**Background:** Congenital disorders of glycosylation (CDG) and NGLY1-CDDG (NGLY1-congenital disorder of deglycosylation) usually represent multisystem (especially neurovisceral) diseases with liver involvement reported in some of them. The aim of the study was to characterize the liver phenotype in CDG and NGLY1-CDDG patients hospitalized in our Institute, and to find the most specific features of liver disease among them.

**Material and Methods:** The study involved 39 patients (from 35 families) with CDG, and two patients (from two families) with NGLY1-CDDG, confirmed molecularly, for whom detailed characteristics of liver involvement were available. They were enrolled based on the retrospective analysis of their medical records.

**Results:** At the time of the first consultation, 13/32 patients were diagnosed with hepatomegaly; none of them with splenomegaly. As many as 23/32 persons had elevated serum transaminases, including 16 (70%) who had mildly elevated levels. During the long-term follow-up (available for 19 patients), serum transaminases normalized in 15/19 (79%) of them, including a spontaneous normalization in 12/15 (80%) of them. The GGT activity was observed to be normal in all study cases. Protein C, protein S and antithrombin activities in plasma were observed in 16 patients, and they were decreased in all of them.

**Conclusions:** It is necessary to conduct a long-term follow-up of liver disease in CDG to obtain comprehensive data.

## Introduction

Glycosylation comprises the main process of the post-translational modification of most human proteins and is critical for physiological and pathological cellular functions. Congenital disorders of glycosylation (CDG) are a heterogeneous group of genetic defects in the synthesis of glycans and their attachment to proteins and lipids. Since the first description of phosphomannomutase 2 deficiency (PMM2-CDG), more than 130 CDG subtypes have been reported (1–3). On the other hand, deficiency of N-glycanase 1 (NGLY1) comprises the only one entity defined as congenital disorder of deglycosylation (CDDG) (4, 5). N-glycanase 1 is a deglycosylating enzyme that cleaves N-glycans from misfolded glycoproteins during the endoplasmic reticulum (ER)-associated degradation (ERAD) process (6).

CDG and NGLY1-CDDG usually represent multisystem (especially neurovisceral) diseases with liver involvement reported in some of them (7). As observed in the severe phenotypes, sometimes associated with non-immune hydrops fetalis, the liver disease was usually part of multiple organ failure, leading to death in the 1st months of life (7, 8). In neurovisceral phenotypes, the liver disease was generally diagnosed based on elevated serum transaminases, independently from the neurological disease (7, 9, 10). However, both liver steatosis and fibrosis (and even cirrhosis) have been reported in the course of some of them (7, 11–13). There are also some subtypes, like MPI-CDG, TMEM199-CDG and CCDC115-CDG, with clinical and biochemical phenotype expressed mainly in the liver (no neurological involvement) (7, 14–19).

Since next-generation sequencing (NGS) became more widely available, an improvement in diagnostics has been observed, with more patients as well as new diseases being reported (1–3). However, the exact data on liver involvement in various CDG subtypes as well as NGLY1-CDDG are sparse and diverse, especially in relation to the long-term follow-up. There is also no general statement on whether the liver disease in various CDG and NGLY1-CDDG is clinically relevant.

The aim of the study was to characterize the liver phenotype in CDG and NGLY1-CDDG patients hospitalized in our Institute and to find the most specific features of liver involvement.

## Materials and Methods

The study involved 39 patients (from 35 families) with CDG, and two patients (from two families) with NGLY1-CDDG, confirmed molecularly. Detailed clinical, biochemical and molecular characteristics were recently published (20–22). All study patients were diagnosed and followed-up in Department of Pediatrics, Nutrition and Metabolic Diseases, Children's Memorial Health Institute, Warsaw, Poland.

The patients for whom detailed characteristics of liver involvement were available were enrolled in the study based on the clinical (physical examination), biochemical (aspartate and alanine aminotransferase, gamma-glutamyl transferase, serum total and direct bilirubin, internal normalized ratio, protein C and protein S activity, antithrombin activity), and histopathological (liver biopsy or autopsy) data.

The reference ranges of GGT in the 1st year of life were in accordance with Cabrera-Abreu and Green (23, 24). The upper limit of normal (ULN) for ALT was modified in accordance with Schwimmer et al. (25).

For the purpose of the study, of the levels of elevation of serum transaminases were divided into the following categories: mild elevation (<3 times of the ULN), moderate elevation (3–5 times of the ULN), severe elevation (>5 times of the ULN).

## Results

### Overall Characteristics

A total of 32 patients were included in the study, including 10 PMM2-CDG, 4 SRD5A3-CDG, 3 ATP6AP1-CDG, 3 MPI-CDG, 3 ALG13-CDG, 3 ALG1-CDG, 1 ALG3-CDG, 1 PGM1-CDG, 1 DPAGT1-CDG, 1 ATPV0A2-CDG, and 2 NGLY1-CDDG.

In patients with MPI-CDG, the clinical and biochemical phenotype was mainly expressed in the liver, whereas in other cases, liver involvement was associated with neurological manifestation/neurovisceral disease (PMM2-CDG, SRD5A3-CDG, ALG13-CDG, ALG1-CDG, ALG3-CDG, DPAGT1-CDG, NGLY1-CDDG), cardiac involvement (dilated cardiomyopathy and progressive cardiac insufficiency in the patient with PGM1-CDG), *cutis laxa* in ATPV0A2-CDG, sensorineural hearing loss, glomerular and tubular dysfunction in ATP6AP1-CDG. One PMM2-CDG patient demonstrated a severe phenotype and succumbed to multiple organ failure at 2 months of age.

The characteristics of clinical, biochemical and histological liver phenotypes are summarized in Table 1.

**Table 1:** Biochemical and histological characteristics of liver disease in study patients with CDG/NGLY1-CDDG defined molecularly.

**CDG/CDDG**	**Mutation and protein change**	**Age at first presentation and during follow-up**	**H/S**	**Laboratory analyses** **Liver biopsy (if performed)** **Other features**
ALG1-CDG	c.773C>T, p.Ser258Leu/ c.1182C>G, p.Phe394Leu	3 mo Death	H (+) S (−)	3 mo – thromobotic event AST 80 ALT 85 INR 1.00 Alb 24 AT 12 Protein C 6.8 Protein S 33
ALG1-CDG	ALG1 c.773C>T, p.Ser258Leu/ c.1182C>G, p.Phe394Leu	1 mo	H (−) S (−)	AST 20 ALT 17 INR 1.15 Alb 21 Protein C 10.6 Protein S 32
		4 mo	H (−) S (−)	AST 23 ALT 9 INR 1.15 AT 15 Protein C 4.7 Protein S 24.4
ALG1-CDG	16p13.3 deletion in the *ALG1* gene	1 mo	H (−) S (−)	ALT 53 AST 49 INR 1.05 AT 30 Protein C 20 Protein S 42
		10 mo	H (−) S (−)	ALT 17 AST 29
ALG3-CDG	n.a.	10 mo	H (−) S (−)	AST 20 ALT 14 INR 0.99 Alb 39 AT 61 Protein C 54 Protein S 51
		10y 5mo	H (−) S (−)	AST 33 ALT 32 INR 0.95 Alb 43 AT 58 Protein C 52 Protein S 65
ALG13-CDG	c.320A>G, p.Asn107Ser, hmz *de novo*	12mo	H (−) S (−)	AST 44 ALT 29 INR 1.00
		4y	H (−) S (−)	AST 27 ALT 5 INR 0.9
ALG13-CDG	c.320A>G, p.Asn107Ser, hmz *de novo*	8mo	H (−) S (−)	ALT 16 AST 33 INR 1.03
		2y	H (−) S (−)	AST 31 ALT 5 Alb 43 INR 0.97
ALG13-CDG	c.320A>G, p.Asn107Ser, hmz *de novo*	6mo	H (−) S (−)	AST 22 ALT 12 INR 1.00
ATP6AP1-CDG (X-linked)	c.1284G>A, p.Met428Ile	8y	H (−) S (−)	AST 70 ALT 76 INR 1.05
		17y	H (−) S (−)	AST 61 ALT 55
		25y	H (−) S (−)	AST 54 ALT 46
ATP6AP1-CDG (X-linked)	c.1284G>A, p.Met428Ile	9y	H (−) S (−)	AST 64 ALT 39
		18y	H (−) S (−)	AST 46 ALT 51
ATP6AP1-CDG (X-linked)	c.1284G>A, p.Met428Ile	30y	H (−) S (−)	AST 68 ALT 52
		36y	H (−) S (−)	AST 57 ALT 48
ATPV0A2-CDG	c.2015T>A, p.Leu672X/ c.130delG, p.N43fsX55	1y9m	H (−) S (−)	AST 69 ALT 23 Alb 45 INR 1.01 Protein C 17 Protein S 38
DPAGT1-CDG	c.1117C>G, p. Pro373Ala/ c.1197T>A, p.Tyr399X	6mo Death	H (−) S (−)	AST 102 ALT 104
MPI-CDG	c.1193T>C, p. Ile398Thr, hmz	2y	H (+) S (−)	AST 196 ALT 365 INR 1.05 Protein C 41.4 Protein S 52.4 Ceruloplasmin 0,3
				Liver biopsy–mild focal foamy degeneration of hepatocytes, without steatosis.
		4y	H (−) S (−)	On mannose supplementation therapy AST 41 ALT 70 AT 50
		9y	H (−) S (−)	On mannose supplementation therapy AST 24 ALT 21 INR 1,01 AT 76 Protein C 110 Protein S 124
MPI-CDG	c.1193T>C, p. Ile398Thr, hmz	12mo	H (+) S (−)	AST 168 ALT 355 ATIII 50 Protein C 52 Protein S 50 Ceruloplasmin 0.24
				Liver biopsy–Mild inflammatory infiltrates, mixed macro- and microvesicular steatosis of hepatocytes, foamy degeneration of hepatocytes.
		2y	H (+) S (−)	On mannose supplementation therapy AST 45 ALT 39
		14y 5mo	H (−) S (−)	On mannose supplementation therapy AST 21 ALT 19 AT 86 Protein C 81 Protein S 105
MPI-CDG	c.656G>A, p.Arg219Glu/ c.748G>A, p.Gly250Ser	12mo	H (+) S (−)	AST 77 ALT 60 AT 48,9 Ceruloplasmin 0.34
		4y 5mo	H (−) S (−)	Protein C 63,5 Protein S 60 AT 69
PGM1-CDG	c.988G>C, p.Gly330Arg/ c.1129G>A, p.Glu377Lys	4y	H (+) S (−)	AST 443 ALT 128
				Liver biopsy–fibrous thin strands between portal tracts, mild inflammatory infiltrates, mixed macro- and microvesicular steatosis of hepatocytes, foamy degeneration of hepatocytes.
		10y	H (+) S (−)	AST 269 ALT145 INR 1.02 Ceruloplasmin 0.16
		18y	H (+) S (−)	On galactose supplementation therapy
				AST 73 ALT 39 INR 1.14 AT 61
SRD5A3-CDG	c.292_293del p.Leu98ValfsX121/ c.292_293del p.Leu98ValfsX121	4mo	H (−) S (−)	AST 167 ALT 186 INR 1.24 Protein C 17 Protein S 38 AT 53
		4y 4mo	H (−) S (−)	AST 44 ALT 54 INR, GGTP—normal
SRD5A3-CDG	c.292_293del p.Leu98ValfsX121/ c.292_293del p.Leu98ValfsX121	6mo	H (−) S (−)	AST 214 ALT 291 INR 1.04 Protein C 34 Protein S 51 AT 39
		7y 8mo	H (−) S (−)	AST 18 ALT 13 INR 1.04 GGTP 30 Protein C 40 Protein S 47 AT 53
SRD5A3-CDG	c.424C>T, p.Arg142X/ c.424C>T, p.Arg142X	4mo	H (−) S (−)	ALT 70 AST 55 INR 1.15 GGTP 30 AT 18.3
		9y	H (−) S (−)	ALT 26 AST 26 INR, GGTP—normal
SRD5A3-CDG	c.424C>T, p.Arg142X/ c.489C>A, p.Tyr163X	1y 1mo	H (−) S (−)	ALT 114 AST 82 INR 1.03 GGTP 24 AT 45 Protein C 40 Protein S 47 Ceruloplasmin 28
				Liver biopsy–Mild inflammatory infiltrates, mixed macro- and microvesicular steatosis of hepatocytes, foamy cell degeneration of hepatocytes
		11y	H (−) S (−)	AST 51 ALT 58 INR, GGTP—normal
		17y 5mo	H (−) S (−)	AST 25 ALT 24 INR, GGTP—normal
PMM2-CDG	c.155T>G, p.Val52Gly/ c.640-23A>G, p.?	6mo	H (+) S (−)	AST 20 ALT 14
		8y	H (−) S (−)	AST 26 ALT 16 INR 0,95 AT 58 Protein C 113,6 Protein S 48
PMM2-CDG	c.422G>A, p.Arg141His/ c.691G>A, p.Val231Met	3mo	H (+) S (−)	AST 150 ALT 85 GGTP 25 INR 1.02
		9mo	H (+) S (−)	AST 90 ALT 22 GGTP 17 INR 1.00
		16y	H (−) S (−)	AST 25 ALT 10 GGTP 12 INR 0.99
PMM2-CDG	c.169G>A, p.Gly57Arg/ c.422G>A, p.Arg141His	5mo	H (+) S (−)	ALT 122 AST 149 GGTP 36 INR 1.02 Protein C 18 Protein S 53 AT 28
PMM2-CDG	c.422G>A, p.Arg141His/ c.484C>T, p.Arg162Trp	8y	H (−) S (−)	AST 26 ALT 13 GGTP 25 INR 1.00 Protein C 68 Protein S 62 AT 89 Ceruloplasmin 0,23
		16y	H (−) S (−)	ALT 17 AST 21 GGTP 15 INR 1.05 Protein C 75 Protein S 65 AT 82
PMM2-CDG	c.357C>A, p.Phe119Leu/ c.422G>A, p.Arg141His	4mo	H (+) S (−)	AST 210 ALT 146 GGTP 40 INR 1.04
		6mo	H (−) S (−)	AST 262 ALT 494 GGTP 32 INR 0.99
		6y	H (−) S (−)	AST 26 ALT 38 GGTP 23 INR 1.12
PMM2-CDG	c.24delC, p.C9AfsX27/ c.385G>A, p.Val129Met	Prenatal - NIHF		
		1mo	H (+) S (−)	AST 100 ALT 76 GGTP INR 0.99
		9mo death	H (+) S (−)	AST 110 ALT 120
PMM2-CDG	c.24delC, p.C9AfsX27/ c.691G>A, p.Val231Met	2mo death	H (+) S (−)	AST 100 ALT 114 INR 1.74
				Post mortem examination—liver cirrhosis, mixed macro- and microvesicular steatosis, cholestasis
PMM2-CDG	c.691G>A, p.Val231Met/ c.640-15479C>T (deep intronic splice site mutation)	2mo	H (+) S (−)	AST 320 ALT 500 INR 1.1 GGTP 20
		3 mo—thromobotic event		
		5mo	H (+) S (−)	ALT 326 AST INR, GGTP—normal
PMM2-CDG	c.422G>A, p.Arg141His/ c.691G>A, p.Val231Met	Prenatal – NIHF		
		2y	H (+) S (−)	AST 149 ALT 134 INR, GGTP—normal
PMM2-CDG	c.710C>G, p.Thr237Arg/ c.691G>A, p.Val231Met	2mo	H (+) S (−)	AST 58 ALT 45 INR 0.91 GGTP normal Alb 23 PLT 94 AT 15
		9mo	H (+) S (−)	AST 82 ALT 76 Alb 35 INR 1,25 AT 35
NGLY1-CDDG	c.1789+1G>A, p.?/ c.1063T>C, p.?	3y	H (−) S (−)	ALT 150 AST 125 GGTP 30 INR 1.05
				Liver biopsy–micro- as well as macrovesicular steatosis, minimal lobular fibrosis, amorphous periodic acid-Schiff staining positive diastases-digested material in the cytoplasm.
		7y	H (−) S (−)	ALT 28 AST 40 GGTP 30 INR 1.02
NGLY1-CDDG	c.250G>T, p.Glu84X/ c.1201A>T, p.Arg401X	1y 5mo	H (−) S (−)	ALT 126 AST 142 INR 1.07 GGTP 88

*CDG, congenital disorder of glycosylation; CDDG, congenital disorder of deglycosylation; H, hepatomegaly; S, splenomegaly; “+”, present; “–” absent; mo, months; y, years; AST, aspartate aminotransferase; ALT, alanine aminotransferase; INR, internal normalized ratio; GGTP, gamma-glutamyltranspeptidase; AT, antithrombin; NIHF, non-immune hydrops fetalis; hmz, homozygote*.

### Clinical and Biochemical Phenotype

At the time of the first consultation, 13/32 patients were diagnosed with hepatomegaly, including 8/10 with PMM2-CDG, 3/3 with MPI-CDG, 1/1 with PGM1-CDG, and 1/3 with ALG1-CDG. None of the persons was diagnosed with splenomegaly. Prolonged neonatal jaundice was not observed in the studied patients.

As many as 23/32 (72%) patients had elevated serum transaminases. Mild elevation was observed in 16 (70%) of them, moderate elevation in three cases (one with PGM1-CDG, one with SRD5A3-CDG, one with PMM2-CDG), and severe elevation in four patients (two with MPI-CDG, one with SRD5A3-CDG, one with PMM2-CDG). During the long-term follow-up (available for 19 persons), serum transaminases normalized in 15/19 (79%) of them, including 3 MPI-CDG patients on mannose supplementation therapy and 12 patients with other CDG and a spontaneous improvement. The same level of transaminase elevation was observed in 3/3 patients with ATP6AP1-CDG and 1/1 with PGM1-CDG.

The GGT activity was observed to be normal in all study patients. One person with PMM2-CDG had an elevated serum total bilirubin concentration with normal serum direct bilirubin.

Coagulation studies demonstrated an elevated INR in 1/30 patients (PMM2-CDG). Protein C, protein S and antithrombin activities in plasma were available in 16 cases, and they decreased in all of them. Two patients (one with PMM2-CDG and one with ALG1-CDG) with decreased plasma protein C, protein S and antithrombin activity developed thrombotic events (see Table 1).

Serum ceruloplasmin was tested in 6 patients (three with MPI-CDG, one with PMM2-CDG, one with PGM1-CDG, one with SRD5A3-CDG) and it was found to have decreased in one of them (PGM1-CDG). Wilson disease was excluded on the basis of low urinary copper excretion.

The prevalence of extra-hepatic manifestations of various CDG in our patients was recently published.

### Histological Phenotype

A liver biopsy was performed in five persons, including two with MPI-CDG, one with SRD5A3-CDG, one with PGM1-CDG, and one with NGLY1-CDDG. In one PMM2-CDG patient, the histopathological analysis comprised liver autopsy samples. Detailed histopathological studies were recently published (21, 23).

The foamy degeneration of hepatocytes and liver steatosis (micro- as well as macro-vesicular) were observed in 4 out of 5 CDG cases. In two of them, liver fibrosis was also found, including stage 4 (cirrhosis in the PMM2-CDG patient) and stage 2 (in the PGM1-CDG patient) according to the Batts and Ludwig classification, respectively. One of them (PMM2-CDG) also suffered from cholestasis with rosette formation around bile plugs. In two patients (SRD5A3-CDG and PGM1-CDG), apart from steatosis, mild inflammatory infiltrates were observed. There was no correlation between the person's age and histopathological features.

In the NGLY1-CDDG patient, the liver biopsy revealed moderate micro- and macro-vesicular steatosis as well as minimal lobular fibrosis. An amorphous periodic acid-Schiff-positive material digested by diastase was present in the cytoplasm.

### Treatment

Four patients underwent specific monosaccharide supplementation therapy for CDG; PGM1-CDG patients were treated with oral galactose, while MPI-CDG patients with oral mannose. The exact data were recently published (20).

In the case of MPI-CDG patients, the clinical and biochemical features improved after the administration of mannose (normalization of serum transaminases, serum transferrin isoform values close to the reference range).

In the PGM1-CDG patient, the final diagnosis was established at the age of 10 years but the galactose supplementation did not start until the age of 16 years. Serum transferrin isoforms (but not serum transaminases) improved after galactose supplementation.

## Discussion

Liver involvement (disease) is observed in about one-third of inherited metabolic disorders such as CDG. It is unique due to the fact that the liver is one of the main areas of N-glycosylation in the body and most of serum glycoproteins are synthesized by liver hepatocytes (1–3).

Marques-da-Silva et al. (7) published a systematic review of the literature concerning liver involvement in CDG. The authors classified CDG based on liver involvement into two main groups: one with predominant/isolated liver involvement (MPI-CDG, CCDC115-CDG, TMEM199-CDG, ATP6AP1-CDG) and the other associated with liver disease (PMM2-CDG, ALG1-CDG, ALG3-CDG, ALG8-CDG, ALG9-CDG, PGM1-CDG).

Based on the results of our study and the literature review, we would like to propose another categorization/statement. In the great majority of CDG, the liver disease is not clinically significant; it manifests itself with hepatomegaly (less often) and mildly elevated serum transaminases (more often) in early infancy/childhood, which normalizes later in life. However, in the case of severe phenotypes (sometimes proceeded by non-immune hydrops fetalis) leading to early death, severe liver involvement is observed as part of multiple organ failure.

There is also a group of CDG, including MPI-CDG, CCDC115-CDG and TMEM199-CDG, in which the disease is mainly expressed in the liver (no neurological manifestation).

From the histological point of view, there is no typical pattern for liver disease in CDG as well as no correlation with the person's age. The most common histopathological finding in our cohort of CDG patients was the presence of liver steatosis. However, liver fibrosis, or even cirrhosis, was reported in PMM2-CDG, TMEM199-CDG, and NGLY1-CDDG.

As far as PMM2-CDG is concerned, liver involvement may occur as two distinct phenotypes. The first one comprises a severe neonatal/infantile liver failure (sometimes proceeded by non-immune hydrops fetalis) associated with an early-onset neurovisceral disease (9, 26–31). The histological examination usually shows signs of fibrosis or even cirrhosis (cholestasis and steatosis, as well) (26–31). The histopathological analysis of the liver in PMM2-CDG patients with neurological and multivisceral form, reported by De Lonlay et al., revealed fibrosis in all (4) patients with cirrhosis in of them (27). Portal fibrosis was also observed based on the histopathological evaluation of the liver in the patient reported by Aronica et al., who died at 1 month of age (28).

This was also clearly showed in our study on the example of one PMM2-CDG patient, who succumbed to multiple organ failure at 2 months of age. Liver autopsy samples revealed cirrhosis, mixed macro- and microvesicular steatosis (75% of hepatocytes), and cholestasis with rosette formation around bile plugs. It is interesting to note that no biochemical features of cholestasis were observed (normal GGT activity, mildly elevated serum total bilirubin concentration with normal serum direct bilirubin concentration).

The latter phenotype constitutes a non-progressive neurological disease with a mildly expressed liver disease in the form of hepatomegaly with elevated serum transaminases, which often improves with age, as observed in 9/10 PMM2-CDG patients (9, 10, 31). This is also consistent with the findings reported in other cohorts. Over time, the liver phenotype has a similar pattern to the neurological disease, which does not progress but even improves. We may only speculate about the cellular mechanisms of liver regeneration/compensation in the course of the disease.

In the study carried out by Witter et al. on 75 patients (61 patients with longitudinal follow-up data), a several-fold elevation of serum transaminases in infancy/childhood with their normalization during the follow-up was observed (10). Liver biopsies were rarely performed, but they usually showed liver steatosis (32–34).

In the case of CDG with the primary neurological phenotype (neurodevelopmental disorders), including ALG1-CDG, ALG3-CDG, ALG13-CDG, and DPAGT1-CDG, liver is affected in a minority of patients (7, 35–41). However, in the case of severe phenotypes (sometimes proceeded by non-immune hydrops fetalis) leading to early death, liver involvement is more commonly observed as part of multiple organ failure.

The liver autopsy conducted in the patient with ALG3-CDG, reported by Sun et al., who died at 19 days of life, showed bile duct plate malformations and moderate to severe steatosis (35).

MPI-CDG is special among others CDG due to the fact that it is mainly expressed in the liver (no neurological disease) and that it may be treated with the oral administration of mannose (42). Hepatomegaly is the most common clinical sign, sometimes associated with splenomegaly as the cause of the development of portal hypertension (42–48). So far, only one patient required liver transplantation due to chronic liver disease with the development of hepatopulmonary syndrome (43). The elevation of serum transaminases is a common abnormality in MPI-CDG, but GGT and serum bilirubin concentrations are often normal. Liver biopsy usually showed liver steatosis and fibrosis (12, 42–48). Congenital hepatic fibrosis (CHF) and ductal plate malformations were also depicted as distinct pathological liver biopsy findings for MPI-CDG (42–48). In the study of Damen et al., it was also reported that CHF may be the only sign of MPI-CDG for many years (12).

The administration of mannose improves the clinical and biochemical outcome (including serum transferrin isoforms); however, patients can still develop progressive liver fibrosis (42, 43, 45, 48).

As far as PGM1-CDG is concerned, liver involvement is observed in the multisystem phenotype (including congenital malformations, cardiomyopathy, variable endocrine and hematological abnormalities and no neurological disease); it has not been described in the muscular form of PGM1-CDG (7, 9, 14–16). Like in other CDG subtypes, elevated serum transaminases were observed in the majority of patients. There are some reports of liver biopsy findings, including steatosis, cholestasis and fibrosis; cirrhosis, however, was not reported (14–16, 49). In the study of Tegtmeyer et al., liver biopsy was performed in five patients and showed signs of steatosis, cholestasis, and fibrosis (16). An increased amount of glycogen was observed in two of these persons, and in one of them, electron microscopy revealed glycogen deposits in hepatocytes. In our previous study on histological and ultrastructural liver involvement in various CDG, significant ultrastructural abnormalities in hepatocytes with anomalies of the endoplasmic reticulum and mitochondria, and the accumulation of glycogen and lamellar deposits in cytoplasm were found in in the PGM1-CDG patient (21).

In the recently published paper on liver manifestations in 39 CDG patients, Starosta et al. found that 48.6% of those persons had elevated values of alanine aminotransferase and 70.3% of them had elevated values of aspartate aminotransferase (13). These parameters mostly increased during the first 5 years of life in most types of CDG (apart from ALG8-CDG, CCDC115-CDG, MPI-CDG, PGM1-CDG, and TMEM165-CDG patients), but they improved significantly afterwards. In our study, we observed a higher proportion of persons with an elevated level of serum transaminases. However, according to Starosta et al. and other authors, it normalized spontaneously in the majority of patients.

Cholestasis seems to be not a characteristic/typical feature of CDG; it was mostly reported in histopathological liver studies (there are no detailed data on the serum bilirubin concentration and gamma-glutamyl transferase activity). In the study of Starosta et al., 4 out of 39 patients had elevated levels of gamma-glutamyl transferase (GGT), including 3 PMM2-CDG cases and 1 PGM1-CDG case. In our study, there were no patients with elevated GGT values, and only 1 PMM2-CDG person was diagnosed with hyperbilirubinemia (normal serum direct bilirubin) with cholestasis in the liver biopsy specimens.

Coagulation abnormalities require a special attention in CDG. They typically affect both procoagulant and anticoagulant factors, in the case of which antithrombin deficiency, protein C and S deficiency, and factor XI deficiency were mainly observed, respectively (1–3, 50–53). Elevated serum transaminases observed in the majority of our CDG patients were associated with the presence of coagulation disorders, including protein C, protein S, and antithrombin deficiency. Similar observations were reported by Starosta et al. (13). However, only two patients developed thrombotic events.

In some of our CDG patients who had an elevated level of serum transaminases, a low level of ceruloplasmin and a lower serum copper concentration (less often), Wilson disease was suspected. Disturbances in copper metabolism in persons suffering from CDG type II, especially V-ATPase deficiencies, were reported in the literature (17–19, 54–56). The accumulation of copper in the liver was described in the case of both TMEM199-CDG and CCDC115-CDG (17–19). Serum copper was low in 10 out of 11 patients with ATP6AP1-CDG reported by Jansen et al. (54). The mechanism of this phenomenon is not known but a partial loss of either or both of the copper transporting proteins ATP7A and ATP7B was raised (18).

Since the first report by Need et al., a total number of 34 patients with a congenital disorder of N-linked deglycosylation have been reported (57). The pattern of liver disease in NGLY1-CDDG seems to be similar to that in CDG. Elevated serum transaminases were reported in the majority of NGLY1-CDDG patients in early childhood and normalized in some of them (4, 5, 57–61). Rios-Flores et al. have recently reported on a 5-year-old patient with NGLY1-CDDG who presented a severe episode of acute liver failure (ALF) (62). This was the the first report of ALF in patients with NGLY1-CDDG. The authors claimed that ALF was a consequence of an impaired mitochondrial function. This observation was supported by previous reports of mitochondrial respiratory chain dysfunction in patients with NGLY1 deficiency (63).

In our recently published paper on NGLY1-CDDG, we suggested that every patient with developmental disability associated with a hyperkinetic movement disorder and alacrimia/hypolacrima should be tested for NGLY1-CDDG; an increase of serum transaminases and of the abovementioned biomarkers are in favor of this diagnosis (58). However, a detailed characteristics on liver involvement in NGLY1-CDDG is impossible due to insufficient data due to lack of follow-up in the majority of cases.

Liver biopsy have been reported in some patients. Among them, liver steatosis, fibrosis and also cirrhosis (two cases) have been described (59–61). One of the patients described by Lam et al. underwent orthotopic liver transplantation at 21 months of age for liver cirrhosis and presumed hepatocellular carcinoma. In some patients, including one of our NGLY1-CDDG patients, liver biopsy revealed a cytoplasmic storage of amorphous material (with staining properties similar to glycogen) or vacuolization in hepatocytes consistent with the storage (5). In our latest article, we put forward a hypothesis that the presence of cytoplasmic storage of amorphous periodic acid-Schiff positive material digested by diastases reflects the storage of misfolded and probably not degraded N-glycosylated proteins (21).

## Conclusions

It is necessary to conduct a long-term follow-up of liver disease in CDG to obtain comprehensive data.

If CDG is suspected, the normalization of serum transaminases, which was observed in the majority of patients during the long-term observation, could diminish the suspicion of CDG.

Cholestasis was not observed as a characteristic feature of CDG.

## Data Availability Statement

The original contributions generated for this study are included in the article/supplementary material, further inquiries can be directed to the corresponding author/s.

## Ethics Statement

The studies involving human participants were reviewed and approved by Ethics Committee of Children's Memorial Health Institute. Written informed consent to participate in this study was provided by the participants' legal guardian/next of kin. Written informed consent was not obtained from the individual(s) for the publication of any potentially identifiable images or data included in this article.

## Author Contributions

PL and AT-S: project administration and writing—review & editing. AT-S: supervision. PL, AB, PS, and AT-S: investigation. PL: writing—original draft. All authors contributed to the article and approved the submitted version.

## Conflict of Interest

The authors declare that the research was conducted in the absence of any commercial or financial relationships that could be construed as a potential conflict of interest. The handling Editor declared a past co-authorship with several of the authors AT-S, PL, and PS.
